# A Temporal Deep Q Learning for Optimal Load Balancing in Software-Defined Networks

**DOI:** 10.3390/s24041216

**Published:** 2024-02-14

**Authors:** Aakanksha Sharma, Venki Balasubramanian, Joarder Kamruzzaman

**Affiliations:** 1 Melbourne Institute of Technology (MIT), Melbourne, VIC 3000, Australia; 2 Institute of Innovation, Science and Sustainability, Federation University Australia, Ballarat, VIC 3350, Australia; joarder.kamruzzaman@federation.edu.au

**Keywords:** SDN, flow fluctuation, deep temporal reinforcement learning, latency minimization, packet delivery ratio

## Abstract

With the rapid advancement of the Internet of Things (IoT), there is a global surge in network traffic. Software-Defined Networks (SDNs) provide a holistic network perspective, facilitating software-based traffic analysis, and are more suitable to handle dynamic loads than a traditional network. The standard SDN architecture control plane has been designed for a single controller or multiple distributed controllers; however, a logically centralized single controller faces severe bottleneck issues. Most proposed solutions in the literature are based on the static deployment of multiple controllers without the consideration of flow fluctuations and traffic bursts, which ultimately leads to a lack of load balancing among controllers in real time, resulting in increased network latency. Moreover, some methods addressing dynamic controller mapping in multi-controller SDNs consider load fluctuation and latency but face controller placement problems. Earlier, we proposed priority scheduling and congestion control algorithm (eSDN) and dynamic mapping of controllers for dynamic SDN (dSDN) to address this issue. However, the future growth of IoT is unpredictable and potentially exponential; to accommodate this futuristic trend, we need an intelligent solution to handle the complexity of growing heterogeneous devices and minimize network latency. Therefore, this paper continues our previous research and proposes temporal deep Q learning in the dSDN controller. A Temporal Deep Q learning Network (tDQN) serves as a self-learning reinforcement-based model. The agent in the tDQN learns to improve decision-making for switch-controller mapping through a reward–punish scheme, maximizing the goal of reducing network latency during the iterative learning process. Our approach—tDQN—effectively addresses dynamic flow mapping and latency optimization without increasing the number of optimally placed controllers. A multi-objective optimization problem for flow fluctuation is formulated to divert the traffic to the best-suited controller dynamically. Extensive simulation results with varied network scenarios and traffic show that the tDQN outperforms traditional networks, eSDNs, and dSDNs in terms of throughput, delay, jitter, packet delivery ratio, and packet loss.

## 1. Introduction

The evolution of the Internet of Things [1], mobile edge computing [2], and big data [3] has produced phenomenal growth in network traffic globally. It has resulted in equipment deployment such as sensors, routers, and switches with more energy consumption. Better infrastructure and traffic control methods are insufficient to ease the traffic load. Many network infrastructures are hardware-based, integrating all the network management functions into the hardware. This causes a delay in applying any new ideas and sometimes the hardware structure needs to be re-designed to fit the new algorithms [3]. Dedicated devices like traffic shapers, load balancers, and QoS mechanisms are deployed in networks to prevent congestion. They regulate data flow, distribute traffic evenly, and prioritize critical applications, ensuring optimal performance and reliability. Often, these network components, such as network controllers, are under-utilized [4,5], with the average utilization being only 30–40%. In addition, this drops again by a factor of three or higher during off-peak hours [4].

A great 3 has been focused on traditional networks, such as re-engineering and dynamic adaptation [6,7]. Still, these networks’ lack of centralized control makes implementing device management and protocol updates challenging. The best way to cope with traditional network issues is to equip networks with intelligent functionality.

SDNs provide various technical benefits, particularly network traffic engineering [8], due to SDNs’ separation of data and the control plane. The advancement in SDNs has embedded intelligent functionality by learning the complexity of network operation. This improves the network energy efficiency and application Quality of Service (QoS) [9,10]. Despite various SDN benefits, they still have some limitations, such as scalability, reliability, and a single point of failure [11,12]. Many existing SDN load-balancing techniques rely on static controller deployment, where controllers are pre-assigned to specific network segments or devices. While this approach provides a straightforward setup, it lacks adaptability to dynamic traffic fluctuations and may lead to imbalanced load distribution among controllers. Multiple controllers [13,14,15] are required to recover from a single point failure and to provide better network performance [16]. However, multiple controllers face placement issues [17], leading to an imbalanced network load [18]. In the existing literature, many researchers have considered load-balancing issues. Still, most existing works consider the static controller deployment in SDNs, avoiding flow variations and traffic bursts, ultimately leading to imbalanced load among controllers and increasing the network latency, load, and overall cost.

Hence, an optimal solution is essential to handle the dynamic and large-scale traffic. The solution should intelligently map the controllers to dynamically deal with the flow fluctuation, which requires a solution to manage the network load on the fly and handle the traffic flow fluctuation. Some advanced SDN load-balancing techniques have been proposed for traffic management, including round-robin load balancing, traffic classification with machine learning, applying unsupervised learning techniques in SDNs, and introducing congestion control methods based on reinforcement learning. These techniques aim to optimize the load distribution by dynamically assigning network segments or devices to controllers based on real-time traffic analysis. However, challenges may arise in efficiently managing dynamic controller mappings and ensuring optimal load balancing without introducing additional overhead.

Additionally, recent advancements in SDN load balancing involve integrating machine learning techniques, specifically reinforcement learning and deep learning. This integration optimizes controller decisions and enhances load distribution efficiency, benefiting from computing technologies like the Tensor Processing Unit (TPU) and Graphics Processing Unit (GPU). These approaches use intelligent algorithms to adapt to varying network conditions and traffic patterns, offering the potential for improved load balancing and network performance. However, managing the dynamic network traffic with flow fluctuation remains a less explored area.

In our prior research work [19], we initially developed reference models for mild, moderate, and heavy network traffic using a standard SDN in traditional networks, followed by proposing and implementing an eSDN [19], which has better QoS than standard SDNs due to the priority scheduling and congestion control algorithms. However, the deployment of controllers in an eSDN is static, which fails to accommodate the growing devices by adjusting their loads in real time. Consequently, it leads to a lack of load balancing among the controllers. To address this limitation, we proposed and implemented a dSDN [19] aiming to sustain the network load with the dynamic flow fluctuation in heterogeneous IoT devices. The dynamic controller mapping in a dSDN enhances the overall network QoS, particularly in a heavy traffic network that emulates the futuristic network. The simulation of the dSDN showed that better performance was observed due to its dynamic controller’s mapping approach for heavy traffic. However, considering the unpredictable and exponential growth of IoT, we expanded the dSDN approach by integrating an agent-based learning technique to align with this futuristic trend.

Thus, this paper introduces an agent-based temporal deep Q learning technique. The temporal Deep Q learning Network (tDQN) functions as a self-learning reinforcement-based model. Notably, this marks the inaugural application of temporal deep Q learning to alleviate the load imbalance with flow fluctuation in the SDN to enhance the QoS. It aims to improve the network latency and Packet Delivery Ratio (PDR) and, thereby, the QoS of applications. Our work in this paper makes the following contributions:

- A multi-objective optimization problem for flow fluctuation is formulated to dynamically divert the traffic to the best-suited controller by proposing a temporal deep Q-learning algorithm in the dSDN;
- We propose a temporal deep Q-learning algorithm in the dSDN environment;
- We demonstrate the tDQN’s high level of success in complex decision-making during traffic bursts, which can maintain an optimized balance among the controllers.

Section 2 presents the related work. The problem formulation is outlined in Section 3. Section 4 proposes the temporal deep Q learning approach. Section 5 provides the implementation of the tDQN and the simulation results. This is followed case study in Section 6 and the conclusion in Section 7.

## 2. Related Works

In recent years, increased efforts have been invested in centralized flow control methods based on SDNs [20,21]. The centralized methods for flow management using a network operating system named NOX are presented in [22,23]. These methods manipulate the switches according to the management decisions. If the incoming packet at a switch matches a flow entry, the switch applies the related actions. A round-robin load-balancing method that uses a circular queue to decide where to send the request of each incoming client is proposed in [24]. It responds to DNS requests with a list of IP addresses. However, these approaches fail to handle real-time traffic fluctuation.

Moreover, centralized re-routing decisions are essential in most mechanisms, but the cost involved in re-routing affects their decision-making efficiency negatively. For instance, the study in [25] is based on load shifting for a data center network when the flow is at its peak. However, this shifting method is not suitable for centralized networks. Another study [26] proposed a capacitated K center approach to identify the minimum number of controllers needed and their position. Still, it is unable to deal with dynamic traffic variation. A comprehensive review of several optimized controller placement problem algorithms in SDNs is addressed in [27], which raised many research challenges, such as unbalanced network load and computing the optimal number of controllers needed in the network.

To address these aforementioned challenges, SDN-based technologies must be applied for network load balancing, traffic forwarding, and better bandwidth utilization [28]. However, the current centralized SDNs cannot handle the IoT dynamic requirements. As a result, available resources are under-utilized in centralized SDNs due to the lack of a dynamic rule-placement approach. Thus, an efficient approach is needed as billions more devices will be connected in the future, generating data exponentially [29]. Therefore, network management is essential to manage the massive collection of information and devices to process the generated data efficiently. The overall network performance depends on resource utilization [30]; thus, under- or over-utilizing network components degrades the network performance and minimizes the network utility. So, suitable technologies are required to control the network traffic flows for load balancing and latency minimization.

Therefore, addressing these challenges is imperative to improve the network’s scalability and robustness. To balance the network load, better architectures must be designed to enhance the network scalability without overloading the controllers. The main aim is to reduce the central component load without reducing the load balance efficiency. In relation to the identified issue, the authors in [31] state that several mechanisms can address the challenge of balancing network load. For instance, one approach involves changing the default controller of a switch by directing all requests from the switch to a new controller, or alternatively, associating each switch with more than one controller. Thus, enabling the switch to send some requests to one controller and rest data to another controller leads to flow distribution; still, this mechanism cannot cope with heavy traffic with flow variation. For a large-scale network, an effective load-balancing algorithm might be required to increase the flexibility of the network.

In many instances, Deep Reinforcement Learning (DRL) has yielded impactful results, demonstrating outstanding performance in various applications, such as natural language processing and games [32]. Beyond this, many businesses have begun using deep learning to enhance their services and products. A machine learning technique based on DRL is well-suited to achieving favorable outcomes. It can explore the vast solution space, adapt to rapid fluctuations in the data flow, and undergo algorithmic learning from feedback [33,34,35]. The study [36] introduced a mathematical model to calculate the number of controllers required in the network and their connection with switches. However, this approach is time-consuming, rendering it ineffective for large-scale networks.

In another study [37], a cluster controller is proposed to facilitate the movement of switches, enhancing network throughput but at the cost of increased controller response time. Based on the dynamic migration of switches using swarm optimization, a different approach is presented in [38], resulting in elevated costs, and often the network remains unstable. An alternative strategy, outlined in [31], considers network load. When any controller becomes overloaded, it randomly selects another load to shift its load but does not consider the scenario where another controller may become overloaded after the migration. The authors in [39] proposed a mechanism utilizing reinforcement learning to balance the network load, but this method proved ineffective in achieving load balance.

The extensive literature on reinforcement learning can be classified as controller optimization and switch migration methods to overcome the issues of unbalanced network load. The controller optimization optimizes the number of controllers needed and the location to place them in the network. At the same time, switch migration methods manage the network load by migrating the load from one controller to another controller. In summary, the existing research only found better solutions for static load balancing and targets only one or two issues related to SDN controllers. No solution in the literature satisfies the network performance, load balancing, latency minimization, and dynamic flow fluctuations. In response to this gap, our novel temporal Deep Q-Network (tDQN) is introduced into the dynamic SDN (dSDN) environment, aiming to improve network Quality of Service (QoS) significantly.

## 3. Problem Formulation

The overall SDN-based network can be seen as a directed graph $G=(c_{i},s_{i},L_{i})$ where $c_{i}$ denotes a set of controllers, $s_{i}$ denotes the set of associated switches, and $L_{i}$ denotes the link between the controller and switches. The switch is used to minimize the average latency of the network and ensure the QoS. The whole latency of a single flow can be expressed as $L_{w}^{c,s}$ in the following equation:

(1) $$L_{w}^{c,s}=t_{sl}+t_{cl}+t_{RTT}$$

where $t_{sl}$ is the latency of the switch, $t_{cl}$ is the latency of the controller, and $t_{RTT}$ is the round-trip time.

Switch latency, $t_{sl}$, is the sum of the delay experienced by the flow in the queue, $t_{q}^{s}$, and the flow processing time, $t_{p}^{s}$. Overall, $t_{sl}$ can be expressed as:

(2) $$t_{sl}=t_{q}^{s}+t_{p}^{s}$$

Similarly, $t_{cl}$ can be expressed as the sum of the time that the controller packets spend in the queue, $t_{q}^{c}$, and the controller flow processing time $t_{p}^{c}$:

(3) $$t_{cl}=t_{q}^{c}+t_{p}^{c}$$

The single-trip time is denoted as $t_{cs}^{STT}$ and is the amount of time it takes for a request to be sent from the controller to the switch. Thus, the round-trip time $t_{RTT}$ is the sum of the time a request takes to be sent from the controller to the switch, $t_{cs}^{STT}$, and from switch to the controller, $t_{sc}^{STT}$. Therefore, $t_{RTT}$ can be written as follows:

(4) $$t_{RTT}=t_{sc}^{STT}+t_{cs}^{STT}$$

The single-trip time from the controller to the switch and the switch to the controller can be estimated using the distance of tracking packet routes $\left(D_{sc}\right)$ and signaling speed $\left(C_{o}\right)$.

Considering this, the single-trip time from the switch to the controller $t_{sc}^{STT}$ can be expressed as follows:

(5) $$t_{sc}^{STT}=\frac{D_{sc}}{C_{o}}$$

As the value of $C_{o}$ is constant and comparable with the speed of light, the only possibility for enhancing the single-trip time is through $D_{sc}$.

Similarly, the single-trip time from the controller to the switch $t_{cs}^{STT}$ can be expressed as follows:

(6) $$t_{cs}^{STT}=\frac{D_{cs}}{C_{o}}$$

We can only make improvements in route tracing. Combining Equations (4)–(6), the total round-trip time can be rewritten as follows:

(7) $$t_{RTT}=\frac{D_{sc}}{C_{o}}+\frac{D_{cs}}{C_{o}}$$

Therefore, from Equation (1), we can calculate the overall delay experience in the network of *N* controllers in the following equation:

(8) $$L_{N}=\sum_{i=1}^{N}L_{wi}^{c,s}$$

where $L_{w}$ is the latency of a single flow, $L_{wi}^{c,s}$ is the total number of links established between the controller and switches, and *i* varies from 1 to *N*, where *N* is the total controllers and $L_{N}$ represents the total latency.

Then, the average latency of the network can be calculated:

(9) $$L_{avg}=\frac{1}{N}L_{N}$$

Our problem can then be formulated as a switch-to-controller assignment and a network dynamic route adaptation problem. While assigning a switch to the controller, the selection should be dynamic and consider the following key points: (i) present load on the switch; (ii) load on the corresponding controller; and (iii) round-trip time depending on the packet tracing path.

The complete state table $S_{t}$ can be summarized as the state of the switch along with its corresponding controller. The state $S_{i}$ can interact with the controller $C_{j}$. Moreover, it can be denoted as follows:

(10) $$S_{t}=\left(S_{i}C_{j},S_{x}C_{y},\ldots \ldots \ldots \ldots S_{h}C_{h}\right)$$

where i,j,x,y⊂P, and i,j,x,y≤h,

– *P* represents the network;
– *h* is the maximum state and controller combinations.

The controller selection, deployment, and switch-controller mapping depend on the actions taken by a software agent placed on switch nodes. The agent acts based on the current state. Therefore, we can summarize that switch actions are combinations of agent actions happening at a single switch node. Each switch has a number of possibilities to redirect the inflow, denoted as $F_{p}$; thus, every flow can reach the $F_{p}$ number of switch nodes.

Assuming a switch node is denoted by $S_{wi}$, *K* represents the combination of possible actions that a switch can have. Thus, the action of each agent, $A_{si}$, can be written as follows:

(11) $$A_{si}=\left(S_{wi}^{1},S_{wi}^{2},\ldots ,S_{wi}^{K}\right)$$

where

(12) $$S_{wi}^{K}=\left\{\begin{array}{cc} 1, & \mathrm{if}\;S_{wi}\epsilon \;C_{j}\\ 0, & \mathrm{otherwise} \end{array}\right.$$

Therefore, combined action space, $A_{c}$, can be presented with all actions taken at a network as:

(13) $$\begin{array}{c} A_{c}=\left\{A_{s1},A_{s2},A_{s3},\ldots ,A_{sK}\right\}\\ \mathrm{where}K\subset F_{p} \end{array}$$

Considering this, the controller’s load cannot exceed its maximum limit. An agent’s action in tDQN is rewarded if its current action favors the overall goal of minimizing network latency in exploring an optimized solution from the possible combinations in the action space. The reward can be defined as a metric of the mean latency of the network as follows:

(14) $$r_{l}={\;}^{◡}(L_{avg})$$

where $L_{avg}$ is defined in Equation (9). The lower the latency, the higher the agent’s reward, and vice versa.

As mentioned above, during the iterative learning process, an agent learns how to make a better decision for switch-controller mapping through a reward–punish scheme to maximize the decision goal by reducing network latency. The details of tDQN are described in the following section.

## 4. Temporal Deep Q-Learning (tDQN)

The tDQN model is based on the principles of reinforcement learning and deep Q-learning. The model dynamically diverts traffic to the best-suited controller based on a multi-objective optimization problem for flow fluctuation. It is an unsupervised learning strategy that can adapt the data without a special mark in the most common datasets and can rapidly be adopted in high fluctuation data with its general feedback mechanism.

The NetSim simulation framework is configured to assess the efficiency of the tDQN model when compared to traditional networks, eSDNs, and dSDNs. Our earlier research [19] presented the simulation setup, and the tDQN is integrated into the dSDN controller within the same configuration. The setup involves creating network scenarios with varying traffic loads, including mild, moderate, and heavy network traffic conditions. Parameters such as throughput, delay, jitter, packet delivery ratio, and packet loss are measured and compared across different network setups. The simulations involve emulating dynamic traffic fluctuations and evaluating the tDQN model’s ability to maintain an optimized balance among the controllers. The most critical factors influencing the performance of the Temporal Deep Q Learning Network (tDQN) in different network environments are as follows:

- The Q-learning is fundamental to the tDQN, influencing its ability to learn and adapt to diverse network scenarios;
- The reward–punishment scheme enables the tDQN to dynamically optimize switch-controller mapping, allowing it to adapt to various network sizes, topologies, and traffic patterns;
- The scalability of the tDQN ensures its effectiveness across different network sizes;
- The tDQN’s adaptability to different network types (traditional networks, SDNs, eSDNs, and dSDNs) is vital.

**Figure 1:**
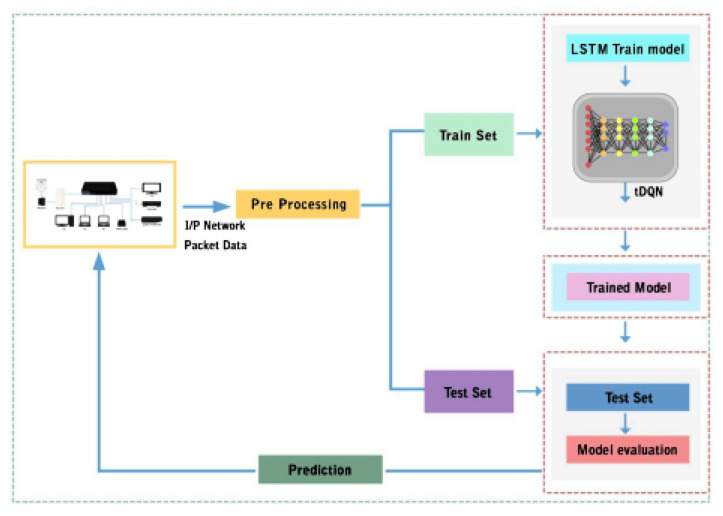
Block diagram of tDQN.

**Figure 2:**
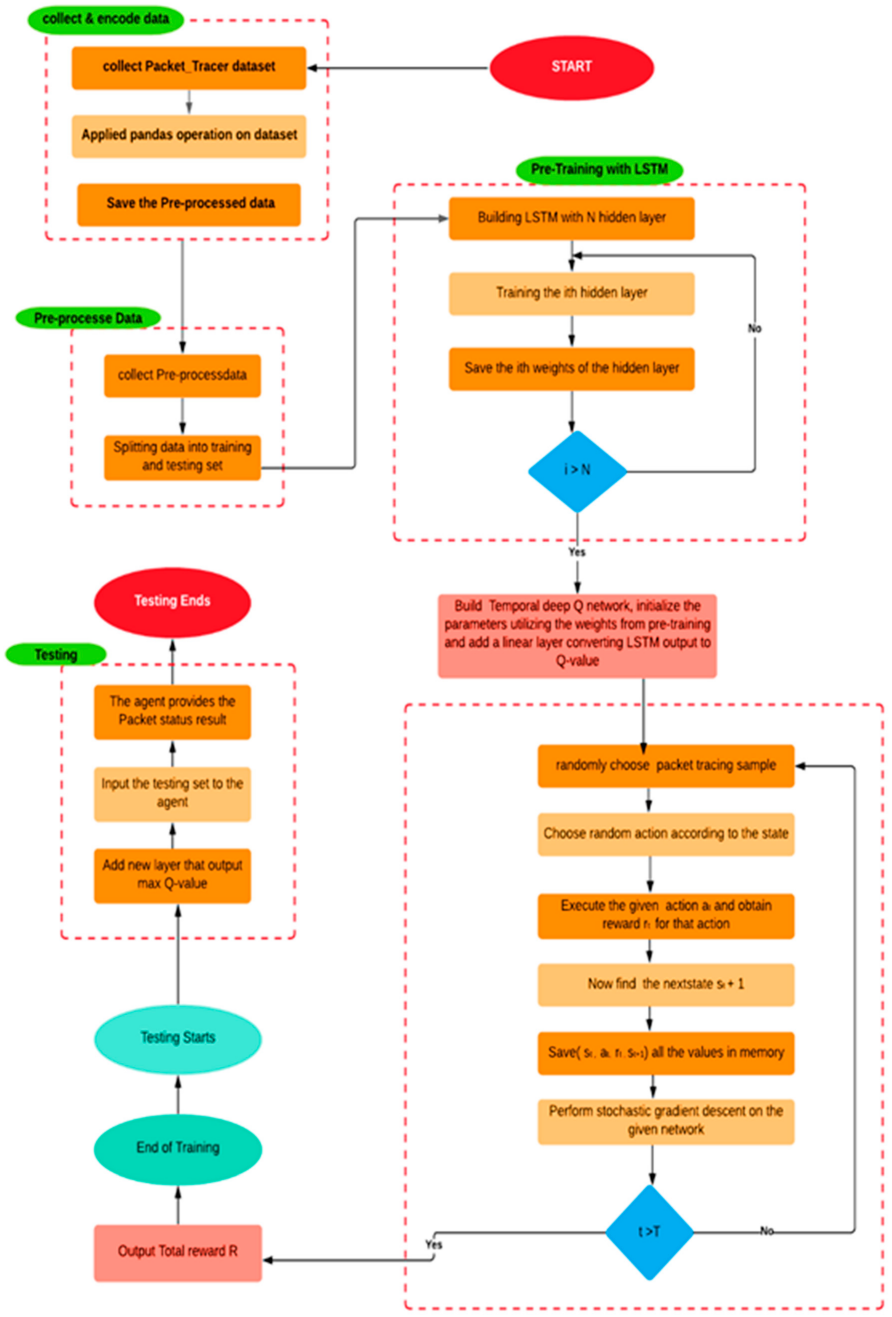
Flowchart of packet tracing in tDQN.

The block diagram for the tDQN is shown in Figure 1. Initially, the data are collected from the network and then pre-processed, followed by training with Long Short-Term Memory (LSTM) and the tDQN. Thereafter, the trained model is tested with test data. Then, the prediction results are sent to the switch for real-time processing. Below is the step-by-step packet tracing process in the tDQN; Table 1 shows the various notations and variables, and Figure 2 shows the flowchart of packet tracing in the tDQN.

### 4.1. Data Collection and Encoding

Figure 2 shows that the process begins by collecting the input variables from the given dataset and applying the Pandas operation [40]. Pandas is a powerful and popular open-source package in Python. It is most widely used for data science, data analysis, and machine learning tasks. It was used to perform data pre-processing. The dataset was selected from the packet tracing files. After visualizing and analyzing the data, it was necessary only to consider the values that impact the target variables. Then, a few functions were performed, such as adding categorical features and filling up the dataset’s missing values. This part was completed by saving the processed data. With this, the pre-processing was complete, and our data were saved as input to the LSTM model.

### 4.2. Data Pre-Processing

This step entails splitting the collected pre-processed data for training and testing purposes. The training data were approximately 80%, while the testing data were 20%.

### 4.3. Pre-Training with the LSTM

In the tDQN, an agent is placed in the SDN controller, which trains itself using an LSTM model. It is framed with the stack of LSTM layers arranged sequentially: the first layer is the input layer consisting of 64 units; the second layer consists of hidden layers, such as the Conv1D layer, which include 64 filters with kernel_size 4 to obtain the tensor output layers, the Flatten layer, which converts the data into a 1D array, and the Dense layer, which feeds all the output from the previous layer to all its neurons. The Dense layer contains two units with a SoftMax activation function. If all the parameters are trained, the model weights for the hidden layer are saved; if not, it is trained again until all the parameters are trained. The last layer is the output layer, which is reached after the training model. We built a tDQN and initialized the parameters by utilizing the weights from the pre-trained model, and a linear layer was added that converts the LSTM output to Q-value.

### 4.4. Training with Q-Learning

Q-Learning is used to train an agent. It trains the agent to learn the mapping from states to actions directly. In Q-learning, a function for the State and Action is defined, representing the maximum discounted future reward when we perform an action in a state and continue optimally from that point. In this case, the Q Function can rate two possible actions that are successful or collided. The agent picks the action with the highest Q-value. Q-values are the action values used in Q-learning to improve the agent’s learning behavior iteratively. A packet tracing sample and an action are selected randomly during the training process. The rewards are obtained based on the executed action defined in (14), and the total output reward is achieved.

### 4.5. Testing

The LSTM can efficiently determine the packet tracing and represent the essential features and its self-learning process. This takes place layer after layer, while the sparse constraints limit the parameter space, which prevents over-fitting. As we added a linear layer that converts the LSTM output to Q-values, the tDQN works dynamically. Once the agent is trained, it can be placed on the controller side and is ready to be used in real-time switches.

### 4.6. Algorithmic Pseudo-Code for tDQN

The pseudo-code outlining our proposed approach is presented in Table 2. The iterative process commences from time step *t* = 1 and continues until the terminal time step or indefinitely. At the start of each iteration, the state $s_{t}$ is obtained from the environment, as depicted in line 1 of the table. Subsequently, the agent selects action $a_{t}$ from the action space based on the state $s_{t}$ using the epsilon-greedy approach, outlined in lines 3 to 8. The epsilon-greedy algorithm aids the agent in deciding whether to explore or exploit. The fundamental concept involves obtaining a Uniform Probability Distribution P on the interval (0,1) and a designated epsilon value ϵ.

In each iteration, a number *p* is selected from this distribution. Line 4 compares *p* and ϵ, and if *p* is less than ϵ, the action will be randomly chosen from the action space using a Uniform Distribution. Otherwise, in line 7, the action $a_{t}$ with the highest estimated reward, denoted *arg*$\max_{a}$Q(s,a,θ), will be assigned to the agent. The *arg*max function identifies the argument that yields the maximum value from a target function. Line 9 executes the action taken by an agent to the environment and observes the reward $r_{t}$ and the next state $s_{t+1}$, referred to as the Sampling Phase. Subsequently, the agent stores the collected transition $\left(S_{t},a_{t},r_{t+1},s_{t+1}\right)$ in the experience replay memory buffer D, as indicated in lines 9 and 10.

Then, line 11 will sample the random mini-batch of N transitions from *D*. Following this, the Learn Phase begins; for each individual transition γ = $\left(s,a,r^{\prime },s^{\prime }\right)$ in the mini-batch, we calculate the target value $y_{r}=r^{\prime }+\gamma$ * $\max_{a^{\prime }}$$Q^{-}\left(s^{\prime },a^{\prime },\theta^{-}\right)$. Then, we compute the Loss function to update the action-value network *Q* using the Gradient Descent algorithm, as detailed in lines 12–16. Gradient descent is used for training machine learning models and neural networks. In line 17, after each M step, we reset the action-value network $Q^{◡}=Q$ to avoid target re-computation.

## 5. Implementation of tDQN and Simulation Results

The tDQN agent was deployed in the dSDN environment to execute intelligent routing, and its performance was evaluated. Several comparisons were conducted for three network scenarios: mild, moderate, and heavy. Initially, the results of the tDQN were compared with the previously proposed dSDN [19] to analyze the network performance. Subsequently, a comparison was made between the tDQN, traditional network, and eSDN. The final comparison encompassed all approaches—traditional networks, SDNs, eSDNs, dSDNs, and tDQNs.

### 5.1. Comparison of dSDN and tDQN

The results obtained from the dSDN and tDQN are elucidated through graphs to facilitate a more comprehensive analysis. The outcomes are succinctly summarized for mild, moderate, and heavy networks in Table 3, Table 4 and Table 5, respectively.

#### 5.1.1. Mild Network Traffic

**Figure 3:**
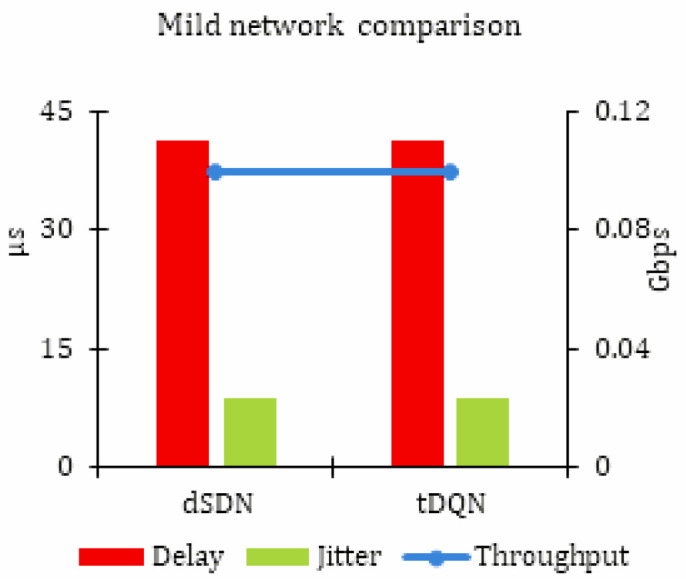
Simulation results for mild network traffic.

Table 3 reveals that there was no substantial change observed when implementing the tDQN in mild network traffic. The results are noted as throughput in Gbps, delay, and jitter in μs for mild network traffic. As this network experiences the least congestion, the efficient performance of the proposed approaches of the eSDN and dSDN is demonstrated. Figure 3 visually presents the results.

#### 5.1.2. Moderate Network Traffic

**Figure 4:**
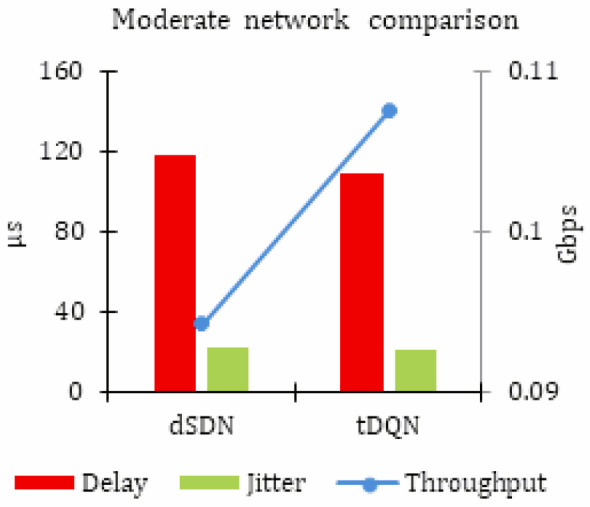
Simulation results for moderate network traffic.

Table 4 summarizes the results for moderate network traffic. The network performance was enhanced using the tDQN. However, as shown in Figure 4, this network was significantly enhanced with the dSDN by increasing network throughput, and reducing delay and jitter. Still, there was a slight increase in network throughput, and a reduction in delay and jitter. The results demonstrate that the proposed tDQN approach provides only marginal enhancements to the Quality of Service (QoS) in this moderate network scenario.

#### 5.1.3. Heavy Network Traffic

Table 5 provides a summary of the results for heavy network traffic, where the proposed tDQN approach stood out as the most effective. The results are noted as throughput in Gbps, delay, and jitter in ms for heavy network traffic. In highly congested network traffic, characterized by increased packet loss, network delay, and jitter, the tDQN demonstrated its capability to enhance the network Quality of Service (QoS). This makes the tDQN particularly suitable for addressing the challenges posed by a growing number of heterogeneous devices in such crowded network environments.

Table 3, Table 4 and Table 5 can be compared to precisely analyze the improvements achieved by integrating the tDQN into the dSDN. Notably, no throughput improvement was observed for mild network traffic. However, for moderate and heavy networks, there was a substantial 14.13% and 20.29% throughput enhancement, respectively.

No significant impact on delay was observed in the case of mild network traffic. However, there was a notable 6.87% reduction in delay for moderate network traffic. The most significant reduction was observed in heavy network traffic, where the tDQN demonstrated an impressive 94.13% decrease in delay.

No significant impact on jitter was observed in the case of mild network traffic. However, a notable 6.50% reduction in jitter was observed for moderate network traffic. The most substantial reduction was noted for heavy network traffic, where the dSDN achieved an impressive 83.49% reduction in network delay.

**Figure 5:**
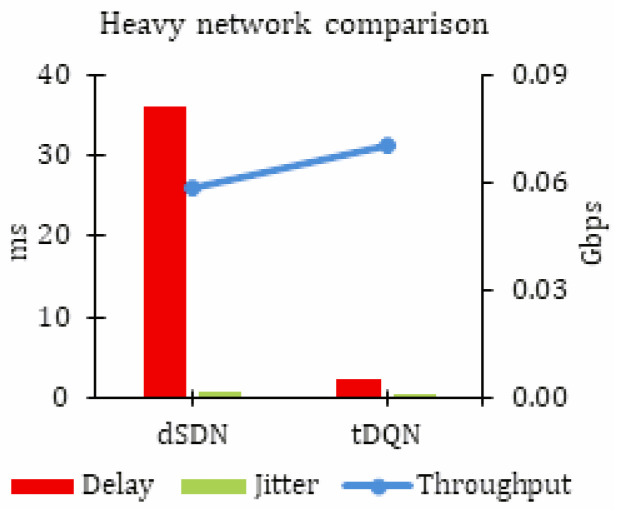
Simulation results for heavy network traffic.

The aforementioned results show that the tDQN proved to be highly beneficial in heavily crowded networks (Figure 5).

### 5.2. Comparison of Traditional Network, eSDN, and tDQN

In this sub-section, the overall simulation results are compared for traditional networks, eSDNs, and tDQNs to analyze the improvement in network QoS. Compared to traditional network management techniques, the tDQN approach offers several advantages. Firstly, the tDQN approach is software-based, making it easier to implement new ideas and protocols without requiring a re-design of the hardware structure. Secondly, the tDQN approach provides centralized control, making it easier to manage devices and implement protocol updates. Thirdly, the tDQN approach utilizes a reinforcement-based model that can adapt to dynamic network traffic and traffic fluctuations, making it more effective in handling real-world network scenarios.

To compare the overall packet delivery ratio and packet loss, we only included the results from the eSDN and tDQN because traditional networks have prolonged packet transmission delays and suffer heavily from QoS degradation.

Table 6 and Table 7 show the overall comparison of traditional networks with the eSDN and the eSDN with the tDQN. The most significant enhancements were evident in moderate and heavy networks. Moreover, these improvements contributed to an overall enhancement in network Quality of Service (QoS), characterized by reductions in delay, jitter, and packet loss. Additionally, there was an observed increase in network throughput and improved packet delivery.

**Figure 6:**
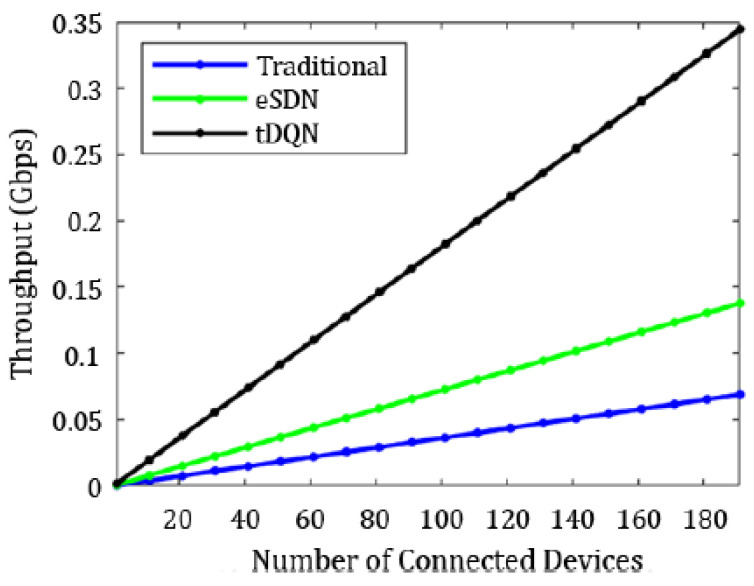
Overall throughput.

**Figure 7:**
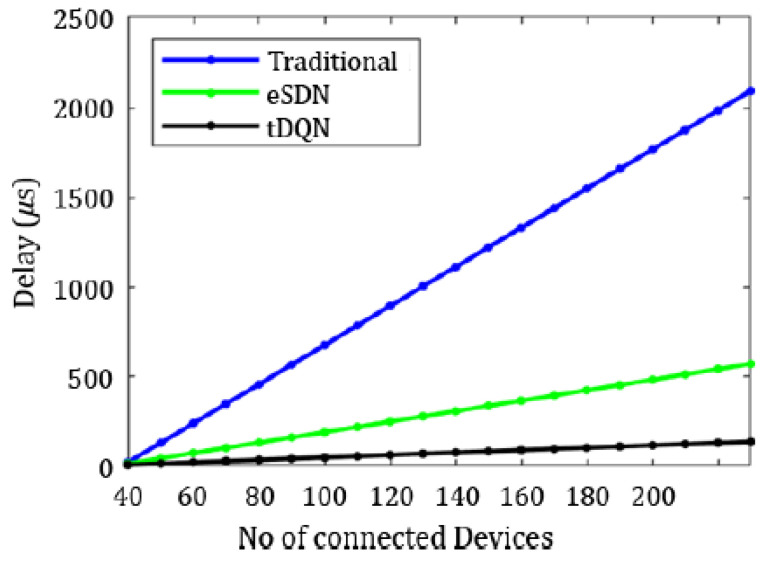
Overall delay.

**Figure 8:**
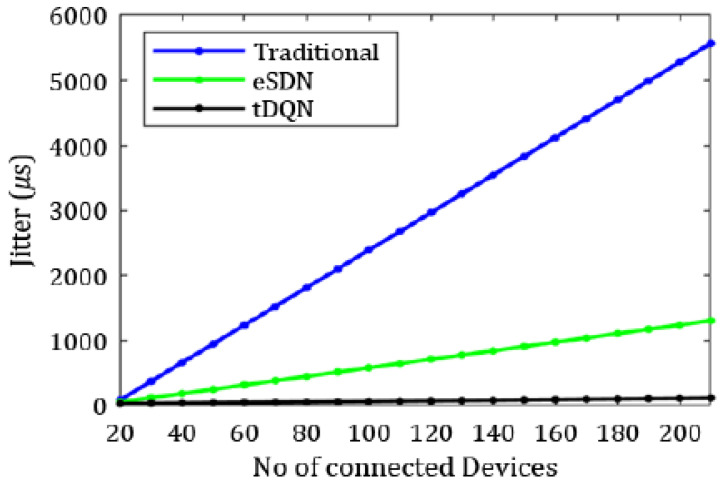
Overall jitter.

**Figure 9:**
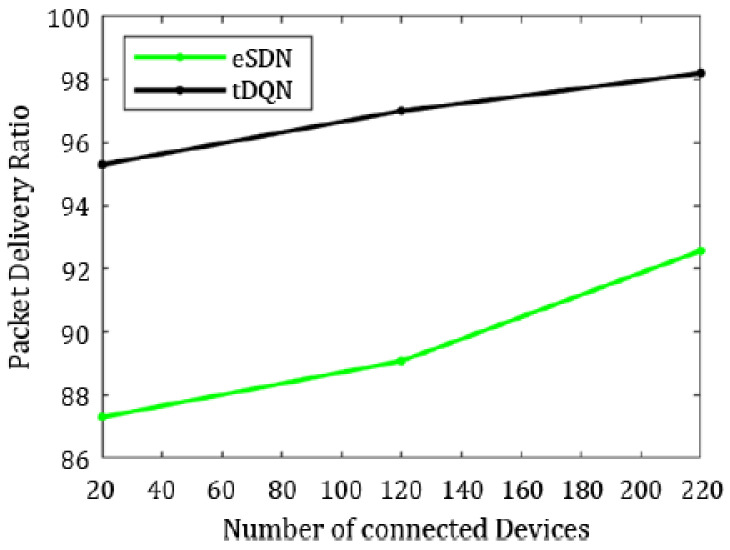
Packet delivery ratio.

**Figure 10:**
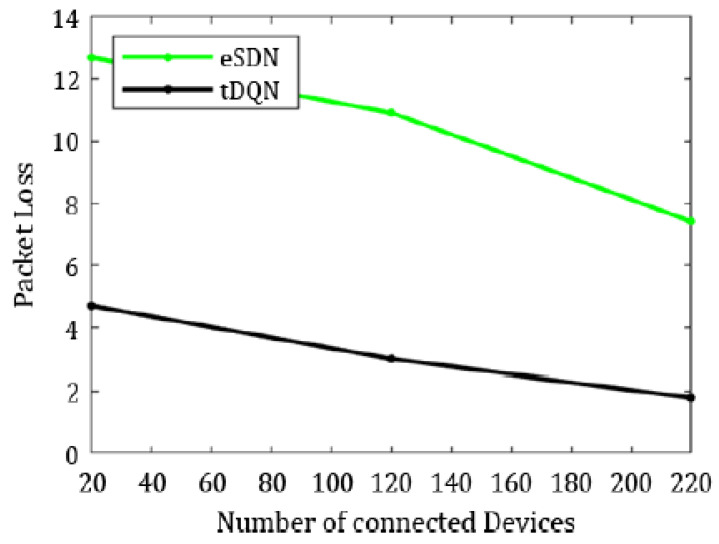
Packet loss.

**Figure 11:**
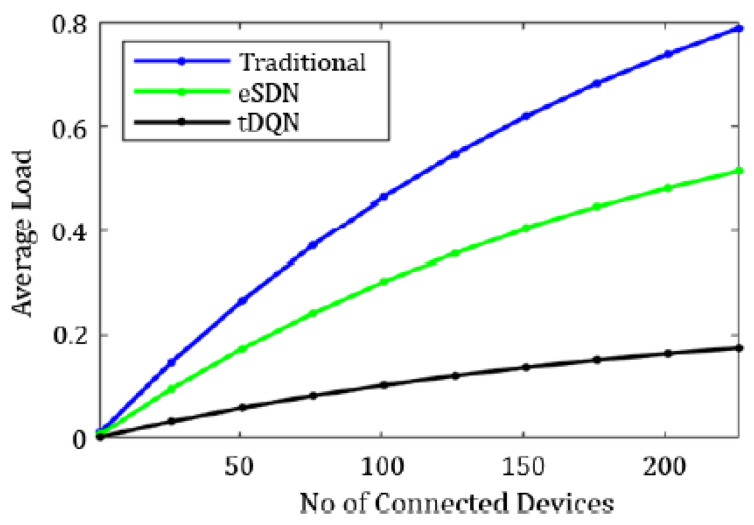
Average load.

Figure 6, Figure 7, Figure 8, Figure 9, Figure 10 and Figure 11 present the plots showing the overall throughput, delay and jitter, packet delivery ratio, packet loss, and average load with an increase in the connected devices for all the networks.

In Figure 6, the increase in throughput is demonstrated using the tDQN in comparison to traditional networks and the eSDN. Moreover, Figure 7 indicates that delay was minimized most effectively with the tDQN, and Figure 8 underscores that jitter reached its minimum using the tDQN. Furthermore, Figure 9 compares the eSDN and tDQN to demonstrate the improvement in the packet delivery ratio using the tDQN. Figure 10 displays the packet loss percentage.

On the other hand, Figure 11 illustrates the average load as the number of connected devices increased in the traditional network, eSDN, and tDQN. In both the traditional network and eSDN, the load distribution remained static, unable to adapt to dynamic traffic fluctuations. Consequently, this resulted in an elevation in the average network load. As the load experienced fluctuations, the static distribution led to an imbalance in the average load, compromising Quality of Service (QoS) standards. In contrast, the tDQN exhibited a lesser spike in the average network load, demonstrating its ability to maintain QoS despite varying network conditions.

The following sub-section compares all the approaches (the traditional network, SDN, eSDN, dSDN, and tDQN).

### 5.3. Overall Comparison of Traditional Network, SDN, eSDN, dSDN, and tDQN

The overall results for the traditional network, SDN, eSDN, dSDN, and tDQN in terms of throughput, delay, and jitter are detailed in Table 8 for mild network traffic, Table 9 for moderate network traffic, and Table 10 for heavy network traffic.

#### 5.3.1. Mild Network Traffic

In Table 8, Table 9 and Table 10, the delay and jitter are considered in milliseconds, while throughput is measured in Gbps. Table 8 illustrates the significant improvements in throughput, delay, and jitter with the implementation of the tDQN, surpassing the traditional SDN and eSDN networks.

Particularly in the least crowded network scenario, the network performance of the tDQN closely aligned with that of the dSDN. The traditional networks and SDN manifested minimum throughput, while the eSDN, dSDN, and tDQN had relatively high throughput. The throughput varied from 0.086 Gbps in the traditional networks and SDN, increasing to 0.100 Gbps for the eSDN, dSDN, and tDQN.

Regarding delay, the traditional networks exhibited the highest delay, gradually decreasing for the dSDN and tDQN. The delay in the traditional network was 0.10 ms, which reduced to 0.07 ms in the SDN, further diminishing to 0.06 ms with the eSDN. Notably, the dSDN and tDQN achieved the same low delay of 0.04 ms.

Similarly, jitter was maximum in the traditional networks and least in the dSDN and tDQN. It reduced from 0.02 ms for the traditional networks to 0.01 ms for the tDQN.

In summary, the tDQN outperformed both the traditional and eSDN networks, demonstrating better network performance. In comparison to the dSDN, the tDQN did not show a significant improvement in network QoS for the least crowded network scenario, as the dSDN already achieved substantial enhancements. However, there was a notable improvement in the packet delivery ratio and packet loss when comparing the eSDN and tDQN. The packet delivery ratio with the eSDN stood at 92.58%, increasing significantly to 98.20% with the tDQN. Furthermore, the findings illustrate a significant improvement in the overall quality of service, with packet loss decreasing from 7.41% with the eSDN to only 1.79% using the tDQN.

#### 5.3.2. Moderate Network Traffic

For the moderate network type, the tDQN outperformed the traditional network, SDN, eSDN, and dSDN. Table 9 presents the throughput, delay, and jitter for all the network types (traditional, SDN, eSDN, dSDN, and tDQN). The traditional network exhibited significant delays and jitter, which were reduced by applying the SDN and eSDN. A further enhancement in the network performance was observed using the dSDN and tDQN.

The throughput varied from 0.078 Gbps in the traditional networks to 0.108 Gbps in the tDQN. The highest throughput was observed with the tDQN, being enhanced by approximately 14.13% as compared with the dSDN. The delay was highest in the traditional networks at 84.25 ms, which reduced to 68.25 ms for the SDN. The most substantial reduction in delay was noted in the dSDN, and it further decreased to 0.11 ms in the tDQN. The delay was reduced by approximately 6.87% in the tDQN compared to the dSDN.

Similarly, jitter was maximum in the traditional networks and least in the tDQN, varying from 0.20 ms in the traditional networks to 0.02 ms in the tDQN. The tDQN jitter was reduced by approximately 6.50% compared to the dSDN. Hence, the tDQN performed better than the traditional network, SDN, eSDN, or dSDN. The packet loss using the tDQN was 2.99%, which is significantly lower than the 10.91% observed in the eSDN. Moreover, the packet delivery ratio using the eSDN was 89.08%, increasing to 97.01% with the tDQN, indicating a notable improvement in the network performance by raising the ratio of successfully delivered packets.

#### 5.3.3. Heavy Network Traffic

The tDQN increased the network performance by reducing delay and jitter for the heaviest network traffic. As indicated in Table 10, the minimum throughput was noted for the traditional networks and SDN, whereas the eSDN, dSDN, and tDQN had a relatively high throughput. The throughput varied from 0.048 Gbps in the traditional networks to 0.070 Gbps in the tDQN.

The delay was highest in traditional networks and minimum in the tDQN. The delay in the traditional network was 235.32 ms, which reduced to 2.12 ms in the tDQN. Similarly, jitter was maximum in the traditional networks and least in the tDQN. It varied from 5.56 ms in the traditional networks to 0.11 ms in the tDQN. A comparison between tDQN and dSDN revealed that the tDQN achieved a 20.29% throughput increase, while delay and jitter were almost eliminated, reducing by 94.13% and 83.49%, respectively, compared to the dSDN. Therefore, the tDQN was able to maintain a high QoS as delay and jitter were significantly reduced despite heavy traffic in the network, whereas the traditional network, SDN, eSDN, and dSDN fell significantly short in this respect.

Ultimately, the tDQN can handle the increasing load on the server. The packet delivery ratio in tDQN was 95.31%, whereas it reached only 87.30% using the eSDN. The packet loss with eSDN was 12.69%; however, it was 4.6% using the tDQN.

Moreover, the tDQN was demonstrated to be a scalable and adaptable solution, as indicated by its reinforcement learning framework, which enables the agent to learn and adapt to different network scenarios. This paper highlights the performance of the tDQN across different network types under varying traffic conditions, such as mild, moderate, and heavy network traffic. The results demonstrate the effectiveness of the tDQN in improving network Quality of Service (QoS) and throughput across these diverse scenarios. The adaptability of the tDQN in real-world network environments is evident in its capability to minimize network latency, optimize packet delivery ratio, and reduce packet loss. These performance metrics play a critical role in various network applications.

## 6. Case Study

The increasing number of IoT devices in a large-scale network can cause network congestion, resource under-utilization, and latency issues. To address these challenges, we propose the implementation of agent-based temporal deep Q learning approach in a dynamic Software-Defined Network (dSDN) environment to optimize load balancing and improve network performance.

This case study focuses on a smart city scenario that involves the deployment of various IoT devices, such as sensors, actuators, and cameras, across different locations within the city. These devices generate a massive amount of data that need to be transmitted, processed, and analyzed in real time to support various smart city applications, including traffic management.

To address these challenges, we propose the implementation of a temporal deep Q learning approach in the SDN controller to optimize load balancing in the network. The temporal deep Q learning algorithm enables the controller to dynamically adjust the routing of traffic flows based on real-time network conditions.

The implementation steps include data collection, training the tDQN model, dynamic traffic routing, and a performance evaluation. By implementing the agent-based tDQN model in the dSDN controller, traffic flows can be dynamically routed to minimize latency and maximize quality of service. The performance of the optimized load-balancing solution can be evaluated in terms of latency reduction, throughput improvement, and enhancement in the packet delivery ratio.

## 7. Conclusions and Future Work

The demand for IoT devices is increasing, and the load on the controller’s side will become quite high in the near future. Accordingly, we propose a temporal deep Q-learning approach as a multi-objective optimization problem solver for flow fluctuation. Through the deployment of an intelligent agent trained to make judicious routing decisions, our proposed method, the tDQN, emerges as a robust solution. The results prove that the tDQN outperforms the traditional SDN, eSDN, and dSDN approaches. Notably, the tDQN stands out by effectively balancing controller loads amidst flow fluctuations, thereby enhancing the network Quality of Service (QoS). This improvement is evidenced through a reduction in latency and a notable enhancement in the packet delivery ratio.

Our proposed tDQN was tested for standard applications such as Email, HTTP, FTP, and video and voice streaming. However, the current network traffic situations do not only depend on these applications. In the future, most critical applications, such as healthcare, will be based on Blockchain technologies. Thus, future studies will test our algorithm for Blockchain applications. Also, training a deep Q-learning network can be computationally expensive, particularly if the network architecture is large or the training dataset is extensive. This may affect the time it takes to train the model before it becomes operational. Our future work will focus on extending this approach and testing it on various applications.

## Figures and Tables

**Table 1:** Notations and variables for tDQN.

Notations	Description
$P_{i}$	Packet ID
$P_{t}$	Packet type
$C_{pt}$	Controlled packet type
$S_{i}$	Source address
$D_{i}$	Destination address
$R_{a}$	Remote hop address
$S_{n}$	Sequence number and queue ID
$A_{ck}$	Destination acknowledgment
$V_{i}$	Maximum prediction probability
*s*	Current state of the environment
$s_{t}$	Current state at time step *t*
$s^{\prime }$	Successive states of the current state *s*
*a*	Action taken by an agent
$a_{t}$	Action taken by an agent at state $s_{t}$
$a^{\prime }$	Successive action taken by the agent at state *s*
*Q*, θ	Action-value network with weights θ
$Q^{-}$, $\theta^{-}$	Target action-value network with weight $\theta^{-}$
*D*	Replay buffer
*B*	Size of each mini-batch
$y_{i}$	Target value for each mini-batch
γ	Discounted factor
ϵ	Epsilon

**Table 2:** Algorithmic Pseudo-code for tDQN.

1	for each time step *t* **do**
2	Receiving state $s_{t}$ from the environment
3	Randomly choosing a number *p* from (0;1) with **Uniform Distribution**
4	if p≤ϵ **then**
5	Choosing $a_{t}$ randomly from *A* with **Uniform Distribution**
6	**else**
7	$a_{t}$ = *arg*$\max_{a}$ Q(s,a,θ)
8	**end if**
9	Observe the transition $\left(S_{t},a_{t},r_{t+1},s_{t+1}\right)$
10	Save the transition to the Replay buffer D
11	Sample mini-batch with size B from the Replay Buffer
12	for each transition γ = $\left(s,a,r^{\prime },s^{\prime }\right)$ in the mini-batch **do**
13	Compute the target value: $y_{r}=r^{\prime }+\gamma$ * $\max_{a^{\prime }}$ $Q^{-}\left(s^{\prime },a^{\prime },\theta^{-}\right)$
14	**end for**
15	Compute the loss: Loss =$\frac{1}{B}\sum_{\gamma }{\left(Q\left(s,a,\theta \right)-y_{r}\right)}^{2}$
16	Make a gradient descent step using $\frac{\delta Loss}{\delta \theta }$
17	After M steps, or *t* mod *M* = = 0, we reset the weights of the network by $Q^{-}=Q$
18	**end for**

**Table 3:** Simulation results for mild network traffic.

Network Type	Throughput (Gbps)	Delay (μs)	Jitter (μs)
dSDN	0.100	41.45	8.79
tDQN	0.100	41.45	8.79

**Table 4:** Simulation results for moderate network traffic.

Network Type	Throughput (Gbps)	Delay (μs)	Jitter (μs)
dSDN	0.094	117.91	21.89
tDQN	0.108	109.81	20.47

**Table 5:** Simulation results for heavy network traffic.

Network Type	Throughput (Gbps)	Delay (ms)	Jitter (ms)
dSDN	0.059	36.08	0.68
tDQN	0.070	2.12	0.11

**Table 6:** Results—Traditional network versus eSDN.

Traditional/eSDN	Throughput Increase (eSDN %)	Delay Reduction (eSDN %)	Jitter Reduction (eSDN %)
Mild	16.77%	40.11%	33.08%
Moderate	20.46%	99.86%	89.16%
Heavy	7.89%	5.26%	76.54%

**Table 7:** Results—eSDN versus tDQN.

eSDN/tDQN	Throughput Increase (tDQN %)	Delay Reduction (tDQN %)	Jitter Reduction (tDQN %)
Mild	0.16%	29.49%	19.08%
Moderate	14.26%	7.50%	7.19%
Heavy	36.69%	99.05%	91.40%

**Table 8:** Simulation results for mild network traffic.

Network Type	Throughput (Gbps)	Delay (ms)	Jitter (ms)
Traditional	0.086	0.10	0.02
SDN	0.086	0.07	0.01
eSDN	0.100	0.06	0.01
dSDN	0.100	0.04	0.01
tDQN	0.100	0.04	0.01

**Table 9:** Simulation results for moderate network traffic.

Network Type	Throughput (Gbps)	Delay (ms)	Jitter (ms)
Traditional	0.078	84.25	0.20
SDN	0.087	68.25	0.15
eSDN	0.094	0.12	0.02
dSDN	0.094	0.12	0.02
tDQN	0.108	0.11	0.02

**Table 10:** Simulation results for heavy network traffic.

Network Type	Throughput (Gbps)	Delay (ms)	Jitter (ms)
Traditional	0.048	235.32	5.56
SDN	0.048	230.95	4.31
eSDN	0.051	222.95	1.31
dSDN	0.059	36.08	0.68
tDQN	0.070	2.12	0.11

## Data Availability

Data was generated in NetSim by configuring various networks.
